# Open Science to Address COVID-19: Sharing Data to Make Our Research Investment Go Further

**DOI:** 10.1007/s43441-020-00250-z

**Published:** 2020-12-24

**Authors:** Névine Zariffa, Jonas Haggstrom, Frank Rockhold

**Affiliations:** 1 NMD Group LLC, Bala Cynwyd, USA; 2 Cytel Corporation, Waltham, USA; 3 Duke Clinical Research Institute, Durham, USA

Over 1000 randomized clinical trials (RCTs) for the treatment and prevention of COVID-19 have been initiated. With access to the data from RCTs, researchers can integrate and summarize findings, evaluate new hypotheses, design future trials, and prioritize the next research questions to be addressed. This ensures that the value from the investment in the RCTs goes beyond the original intent of the trial protocols. None of this is possible without first having easy and responsible systems to allow access to data: the primary tenets of the open science FAIR principles dictate a proactive intent to share results and patient data from clinical trials [Wilkinson]. While much has been written and progress has been made, there is more to be done in this journey to true openness [Rockhold]. Reasons for this include (1) the well-known complexities of data access (patient privacy, content of the trial’s informed consent and the primary data holder’s decision rights as to sharing), (2) concerns about mis-interpretation of data in the context of secondary research (beyond the original intent of the trial), and (3) the use of platform trials where multiple intervention arms are studied relative to a single control arm.

The International COVID-19 Data Alliance (*ICODA*) is one of the groups initiating concerted data sharing as a powerful mechanism to address COVID-19. We focus our attention to RCTs recognizing that the Alliance will encompass many other data types.

1. Access to data. Protocols for release of data from RCTs to an open-research platform have typically taken the form of controlled de-identification algorithms and review mechanisms of research proposals and results to ensure there is no unintended re-identification, both of which can take substantial time. The Alliance is implementing a faster approach based on a Data Dictionary of summary-level data (SLD) that includes harmonized provision of covariates (for subgroup assessments) and specified endpoints at multiple timepoints for all selected trials [https://icoda-research.org/wp-content/uploads/2020/11/Data-Harmonization_pdf.pdf]. The focus on SLD data facilitates the release of data as the risk of re-identification is nil. Use of a common Data Dictionary enables quick and comprehensive analyses across all selected trials.
2. Reliable results. The Alliance has established a statistical expert group which has created visual analytic and statistical analysis tools with embedded safeguards. Best practice workshops are a condition of accessing the data as is an approved research proposal. Researchers can indicate if they require data science or statistical support. Individual researchers are introduced to others with similar research questions to facilitate collaborations. Last, a review panel is engaged prior to release of the results. Not all RCTs will be represented in the Alliance’s platform, so we are including a digitized curated aggregator to provide summary-level data from all publicly available data sources.
3. Platform trials are an efficient use of research resources using adaptive elements: control arms evolve as standard of care changes, new test treatments are allowed to enter the trial over time, and test treatments graduate from the trial once there is clarity about their effect. There is no established approach to sharing of data from an ongoing platform trial. We believe that doing so through SLD as described above once a treatment arm has graduated from the trial is a good compromise to allow secondary research for that treatment/control arm combination while maintaining the trial integrity.

We differentiate from other data sharing platforms who either are not open access (e.g., Transcelerate) or that focus uniquely on patient-level data (e.g., Vivli). ICODA offers both direct and indirect data sharing modalities which provide data researchers with a central view into all available data while the data contributor can determine whether to delegate access control to ICODA or retain it.

Having discussed the Alliance’s current approach to responsible data sharing for RCT data, we turn to recommendations of new trials for vaccines and for treatment of COVID-19.

## Treatment Trials

- Data research based on secondary use of available data collected in RCTs is valuable as demonstrated in the Ebola epidemic. Driving toward good practice is essential to ensure results are both quick and scientifically reliable. Having a high-level single point of contact for requests is a great benefit as is quick escalation to senior decision makers who can adjudicate competing views within their organization.
- Further thought and approaches are needed to balance the information/privacy ratio, recognizing patients who are likely to want their data shared [Mello]. One such approach is that of synthetic data [Raghunathan], and another is giving patients their own voice in the matter.
- It is critical that researchers collaborate. Small underpowered trials are not productive, and worse and can be unethical and misleading when viewed on their own. The answers often lie in the collective body of information. Investigators could join others (e.g., through COVIDCP.ORG or the NIH ACTIV Programme). Collaboration and healthy debate among data researchers advances the methodology and confidence in the results based on secondary use of data.

## Vaccine Trials

The following will maximize the investment made into the vaccine efforts and help us engineer the data structures from the start:

- Common Data Elements. Defining the core set of data elements for clinical trials and embedding these in all trials will enable seamless creation of aggregated data sets for future interrogation.
- A bridge to these Common Data Elements can be created in Real World Evidence data systems. This would allow the continued evaluation of the vaccines in the intended wide-scale real-world use.
- A clear commitment from vaccine developers to rapid data sharing in a common platform. Without the public being sufficiently informed to agree to vaccination, approved vaccines will not have the desired impact on the pandemic.

For both sets of upcoming trials, having clear onward data sharing incentives as part of funding research is essential. This includes consideration of sharing data beyond the initial data repository—to avoid undesirable entanglements should future research question be best served by direct aggregation or integration. Adoption of accepted data standards such as the Clinical Data Interoperability Standards Consortium (CDISC), the Medical Dictionary for Regulatory Activities (MedRA), and the World Health Organisation’s drug dictionary (WHO drug) is an accelerator for future data sharing. Funders have an essential lever in both regards and can institute such elements as conditions of grants. Trialists have a vested interest in maximizing the value from their hard work and investment. Most importantly, the urgent needs of the world’s population require us all to improve our mechanisms for sharing of data and generating actionable insights.
